# Preferences for Technology-Mediated Behavioral Lifestyle Interventions With Different Levels of Coach and Peer Support Among Latino Men: Comparative Study Within One Arm of a Randomized Controlled Trial

**DOI:** 10.2196/29537

**Published:** 2022-02-04

**Authors:** Lisa G Rosas, Nan Lv, Lan Xiao, Kristen MJ Azar, Steven P Hooker, Elizabeth M Venditti, Megan A Lewis, Patricia Zavella, Jun Ma

**Affiliations:** 1 Department of Epidemiology and Population Health Stanford University Palo Alto, CA United States; 2 Department of Medicine University of Illinois Chicago, IL United States; 3 Sutter Health Palo Alto, CA United States; 4 College of Health and Human Service San Diego State University San Diego, CA United States; 5 Department of Psychiatry University of Pittsburgh Pittsburgh, PA United States; 6 Department of Epidemiology University of Pittsburgh Pittsburgh, PA United States; 7 RTI Seattle, WA United States; 8 Latin American and Latino Studies University of California Santa Cruz Santa Cruz, CA United States

**Keywords:** Latino men’s health, technology-mediated behavioral interventions, weight management, mobile phone

## Abstract

**Background:**

Although Latino men have the highest prevalence (45%) of obesity among all men in the United States, traditional weight loss interventions have not effectively engaged this hard-to-reach and diverse group. Offering choices among technology-mediated weight loss interventions may offer advantages.

**Objective:**

The aim of this study is to examine Latino men’s preferences among 3 weight loss intervention options. We also examined whether attendance in group sessions (videoconference and in person) and weight loss differed according to intervention choice.

**Methods:**

Latino men (n=200; mean age 47.3, SD 11.8 years) participated in a comparative effectiveness trial based on primary care and were randomized to receive the 1-year HOMBRE (Hombres con Opciones para Mejorar su Bienestar para Reducir Enfermedades Crónicas; English translation: Men With Options to Improve Their Well-being and Reduce Chronic Disease) intervention. HOMBRE is a weight loss intervention that offers 3 delivery options. During an orientation session, a trained bilingual coach helped men select 1 of the 3 intervention options that differed in coach, peer support, and available language. We used canonical discriminant analysis to assess multivariate associations of demographic, clinical, employment, cultural, and technology use and access factors with men’s intervention choices. We used generalized linear models to estimate weight loss at 6, 12, and 18 months for men in each intervention option.

**Results:**

Among Latino men, 28% (56/200) chose videoconference groups, 31% (62/200) chose web-based videos, and 41% (82/200) chose in-person groups. The canonical discriminant analysis identified 1 orthogonal dimension that distinguished between men who chose an in-person group and men who chose web-based videos. Men who were older, spoke Spanish, and did not use a computer frequently had a higher probability of choosing in-person groups versus web-based videos. For men who selected a group delivery option, 86.9% (107/123) attended ≥25% of the sessions, 83.7% (103/123) attended ≥50% of the sessions, and 73.2% (90/123) attended ≥75% of the sessions, with no differences by type of group (videoconference or in person). Men who chose videoconference and in-person group sessions lost significantly more weight at 6 months (both *P*<.001) and 18 months (*P*=.02 and *P*=.04, respectively) than those who chose web-based videos. Men who chose in-person group sessions also lost significantly more weight at 12 months (*P*=.008) than those who chose web-based videos.

**Conclusions:**

There were significant differences according to demographic, employment, cultural, and technology use factors between men who chose 1 of the 3 intervention options. Men who chose one of the group-based options (videoconference or in person) lost significantly more weight than those who chose web-based videos. Providing options that accommodate the diversity of Latino men’s preferences is important for increasing engagement in behavioral interventions.

**Trial Registration:**

ClinicalTrials.gov NCT03092960; https://clinicaltrials.gov/ct2/show/NCT03092960

## Introduction

### Background

Obesity is a major contributor to the leading causes of death among all men in the United States, such as heart disease, cancer, and type 2 diabetes [1,2]. As part of the largest minority group in the United States, Latino men are disproportionately represented among men with obesity compared with all other races and ethnicities [3]. The US Preventive Services Task Force recommends intensive behavioral lifestyle interventions for obesity treatment [4]. However, research to identify effective behavioral lifestyle interventions has derived primarily from research on non-Hispanic White women [5]. Minority men and Latino men specifically have been particularly underrepresented in this research [5]. Thus, it is imperative to develop and scale interventions tailored to Latino men.

Technology, including web-based and smartphone apps, offers opportunities for extending the reach and engagement of behavioral lifestyle interventions to priority populations such as Latino men. Technology can help overcome prevalent barriers to engaging in interventions because of competing priorities from family, inflexible work schedules, and unreliable transportation. In addition, technology-mediated approaches became essential during the COVID-19 pandemic, when in-person meetings were not allowed or encouraged. Latinos increasingly have access to technology in general and smartphones specifically, which makes this a promising approach to maximize reach and engagement in this population [6-9]. However, little is known about Latino men’s preferences for intervention delivery formats, especially those using technology [10].

### Objectives

The HOMBRE (Hombres con Opciones para Mejorar su Bienestar para Reducir Enfermedades Crónicas; English translation: Men With Options to Improve Their Well-being and Reduce Chronic Disease) trial was designed to compare a culturally adapted behavioral lifestyle intervention for Latino men with minimal-intensity control. The culturally adapted behavioral lifestyle intervention offered men 3 options for engaging in the intervention sessions: coach-facilitated group sessions using web-based videoconferencing, prerecorded videos of group sessions available on the web, and coach-facilitated group sessions in person. The 3 choices differed in the used technology, the level of coach and peer support, and language options. The goal of this study is to examine Latino men’s preferences among the 3 intervention options according to demographic, clinical, employment, cultural, and technology use and access factors. We also examined whether attendance (for the videoconference and in-person groups) and weight loss differed among the intervention options. Understanding Latino men’s preferences according to key baseline characteristics can inform future implementation of technology-based interventions for this high-priority population.

## Methods

### Study Design

The institutional review board for Sutter Health, Northern California, and Stanford University approved the study. All participants provided written informed consent. The trial protocol has been previously published [11]. Participants’ deidentified study data and identifiers were protected following the Protection of Human Subjects protocol. Study recruitment and intervention were not affected by the COVID-19 pandemic as they were completed before the pandemic.

### Recruitment and Participants

A total of 424 Latino men who had a BMI of ≥27 kg/m^2^ and ≥1 cardiometabolic risk factor (high waist circumference, high triglycerides, high blood pressure, high fasting plasma glucose, or low high-density lipoprotein cholesterol) were enrolled in the HOMBRE trial following a multistep process, as described in the trial protocol [11]. Patients with significant psychiatric (eg, bipolar or psychotic disorder) or medical comorbidities (eg, active cancer or organ failure) were excluded. Participants were randomly assigned to receive the 12-month HOMBRE behavioral lifestyle intervention adapted for Latino men (212/424, 50%) or a minimal-intensity intervention (212/424, 50%). This study included men who participated in the HOMBRE intervention and attended an orientation session to make a choice on intervention delivery (200/424, 47.2%). Of the 212 men assigned to the HOMBRE intervention, 200 (94.3%) attended an orientation session and made a choice on intervention session delivery.

### Description of the Intervention

The HOMBRE intervention was based on the Group Lifestyle Balance (GLB) intervention, a group-based adaptation of the original Diabetes Prevention Program intervention [12-14] grounded in social cognitive theory [15]. Social cognitive theory emphasizes a triadic, reciprocally deterministic relationship between the individual, environment, and behavior. The year-long HOMBRE intervention included 12 weekly sessions during the intensive phase (months 1-3) and 8 monthly contacts during the maintenance phase (months 4-12) either by phone or email. The HOMBRE intervention offered men 3 options for engaging in the 12 weekly sessions: coach-facilitated group sessions using web-based videoconferencing, prerecorded videos of group sessions available on the web, and coach-facilitated group sessions in person (Table 1). A trained health coach facilitated the group sessions on videoconference and in person using a cultural adaptation of the GLB. The Latino Patient Advisory Board adapted the GLB and made the following major changes: (1) added an orientation session before session 1 to provide a brief overview of the intervention to participants and family members; (2) incorporated the *MyPlate* visual in the orientation and in the early intervention sessions, given its effectiveness for communicating the types of food choices recommended by the intervention; and (3) invited family members to sessions 6 and 12, given the cultural importance of family support [16]. The prerecorded videos did not include any of these adaptations, except that health coaches encouraged men to watch the videos with a family member. In all 3 options, health coaches encouraged men to self-monitor weight using a study-provided digital scale, physical activity using a study-provided wearable activity tracker, and dietary intake using MyFitnessPal web or a smartphone app (available in Spanish and English).

**Table 1:** Session delivery options in the HOMBRE (Hombres con Opciones para Mejorar su Bienestar y Reducir Enfermedades Crónicas) intervention.

Characteristics	Videoconference	Web-based videos	In person
Description	A bilingual, bicultural coach facilitated weekly sessions on a videoconferencing platform (Zoom)	Men were given access to prerecorded web-based videos of coach-facilitated group sessions	A bilingual, bicultural coach facilitated weekly sessions at the clinic where men were recruited
Coach support	Coach-facilitated sessions Feedback from the coach on diet and physical activity monitoring	Self-directed sessions Option to contact the coach for feedback on diet and physical activity monitoring	Coach-facilitated sessions Feedback from the coach on diet and physical activity monitoring
Peer support	Support from other members of the group	No peer support	Support from other members of the group
Frequency of sessions	Weekly	Self-paced; weekly recommended	Weekly
Language	English or Spanish per preference	English with Spanish subtitles	English or Spanish per preference

In addition to the differences in delivery, the 3 intervention options differed in level and type of coach feedback (Table 1). The videoconferencing and in-person options had the highest level of *real-time* coach involvement. Using the tracking data from participants, the health coach provided individualized feedback on diet and physical activity goals during the intensive phase. Individualized feedback provided ample opportunity for tailoring based on cultural and other individual differences. During the maintenance phase, men who chose the videoconferencing and in-person options received monthly phone calls from the coach, which focused on supporting continued goal progress and problem solving for encountered barriers. The web-based video format had the lowest level of coach involvement, according to the protocol, as participants watched prerecorded videos of a coach facilitating the 12 sessions with a multiethnic group of men and women. Men in this option received standardized weekly messages in months 1 to 3, with reminders to watch the videos, use written materials, self-monitor, and reach out to their assigned coach with questions or requests for more individualized feedback. This was in alignment with this intervention choice, which included less proactive coach interaction. In months 4 to 12, men in the web-based video option received monthly standardized messages that included handouts on maintenance topics, reminders to self-monitor, and contact information for the coach.

### Intervention Choice

Following randomization, all participants attended an in-person group orientation session. Trained health coaches offered the orientation sessions in English and Spanish at different times of the day throughout the 2 weeks following randomization to accommodate all participants. Group sessions were designed for approximately 10 participants but could accommodate varying sizes as needed. Participants were encouraged to bring their partners and other family members to the orientation session with the purpose of increasing understanding and social support among family members. Group orientation sessions followed a standardized protocol that featured a didactic component to provide information on the background and goals of the intervention and a small group discussion component specific to their randomization arm. The goal of the small group discussion for the HOMBRE arm was to support men in making a choice among the 3 intervention options. The small group discussion included 3 components to support men in making a choice: (1) description of the 3 intervention choices provided by a health coach, (2) a worksheet that helped men reflect on each of the 3 options, and (3) a small group discussion on the pros and cons of each option. The worksheet prompted men to think about whether they liked participating in groups, the degree to which they would like support from a coach, their comfort with new technologies, and their availability for attending regular weekly sessions. In the small group, the coach asked men to discuss the pros and cons of each option to assist them in considering the choices. At the end of the session, men were asked to make their choice, given their intervention materials, and assisted with using the activity tracker and other technologies based on their choice.

The intervention participants’ initial choices of intervention delivery were used to group them into the 3 options (videoconference, web-based videos, and in person). The participants were allowed to change their choice within 4 weeks. However, none of the participants elected to change. Coaches could transfer patients from the videoconference or in-person groups to the web-based videos if they did not attend the first 4 sessions; 15 men (n=11, 73% from the videoconferencing group and n=4, 27% from the in-person group) were transferred to the web-based videos group.

### Measures

Baseline characteristics included demographic (eg, age, income, education, marital status, and household size) and clinical characteristics (eg, weight, waist circumference, blood pressure, depression symptoms, quality of life, and sleep function), employment (eg, employment status and occupation), cultural characteristics (eg, language, acculturation, and health literacy), and technology use and access. Weight, waist circumference, and blood pressure were measured in duplicate according to standard protocols [17-19]. Depression symptoms were measured using the 9-item Patient Health Questionnaire, with scores between 0 (best) and 27 (worst) [20,21]. Quality of life measures included the health-related quality of life EuroQol 5-dimension questionnaire [22,23], scored on 5 levels (no, slight, moderate, severe, or extreme problems) for 5 domains, and obesity-specific quality of life, with higher scores (range 0-100) indicating more obesity-related psychosocial problems [24,25]. Sleep function was measured using the Patient-Reported Outcomes Measurement Information System sleep disturbance and sleep-related impairment questionnaires [26]. Sleep disturbance and sleep-related impairment T scores ranged from 25 (high disturbance or impairment) to 80 (none at all), with a mean score of 50 (SD 10) representing the average of the calibration sample, which was generally more enriched for chronic illness. The level of acculturation was assessed using the Short Acculturation Scale for Hispanics, with higher scores (range 1-5) indicating higher acculturation to US society [27]. Health literacy was assessed using the newest vital sign, which uses a food nutrition label, with higher scores (range 0-6) indicating higher health literacy [28]. Technology use and access were assessed using a survey adapted from the Pew Hispanic Trust on technology access and use [29]. Session attendance was recorded in the videoconference and in-person groups only.

### Statistical Analysis

#### Overview

We used 2 steps to identify the different profiles of demographic, clinical, employment, cultural, and technology use and access characteristics based on their choice of delivery options. First, we performed a bivariate analysis to choose a set of candidate variables. Second, we conducted multivariate analysis based on the variables identified in the bivariate analysis to derive the baseline characteristic profiles that significantly differentiated the men who chose 1 of the 3 intervention delivery options [30].

#### Bivariate Analysis

Percentages and means and SDs were used to describe the baseline characteristics among HOMBRE intervention participants overall and by intervention delivery option chosen. We used the Fisher least significant difference method [31] to test for significant differences among all 3 options, which included 2 steps. First, we used analysis of variance (for continuous variables) and chi-square tests (for categorical variables) to compare overall differences in demographic, clinical, employment, cultural, and technology use and access factors across the 3 intervention delivery options. Second, variables with *P*<.05 (2-tailed) from the first step were then further assessed for pairwise comparisons using Student *t* tests for continuous variables and chi-square tests for categorical variables.

#### Multivariate Analysis

Canonical discriminant analysis was used to derive linear combinations of the baseline characteristic profiles that significantly differentiated the men who chose 1 of the 3 intervention delivery options. Canonical discriminant analysis is a multivariate dimension reduction technique that derives a linear combination of explanatory variables that has the highest possible multiple correlation with the groups of a classification variable. The dimension defined by the linear combination is the first canonical dimension. This maximum multiple correlation is called the first canonical correlation. The coefficients of the linear combination are the canonical coefficients. The second canonical dimension is obtained by finding the linear combination with the next highest possible multiple correlation with the groups that is uncorrelated with the first canonical dimension. The process of extracting canonical dimensions can be repeated until the number of canonical dimensions equals the number of original variables or the number of groups minus 1, whichever is smaller. We included only the baseline characteristics with *P*<.15 from the bivariate analyses [32]. The categorical variables were coded as dummy variables in the canonical discriminant analysis. Standardized canonical coefficients measured the strength and direction of the correlation of each dimension with the characteristics. Participant scores on each dimension were calculated as the sum of the products of the canonical coefficients and the participant’s individual values for the characteristics. These scores were then compared among the 3 intervention delivery options using analysis of variance.

We used generalized linear models to compare weight loss among men in different intervention options at 6, 12, and 18 months after randomization. We examined weight according to the men’s initial and final intervention choices. Weight was measured by trained study staff at baseline. Weight was measured using a standard calibrated scale at baseline and 18 months at local clinic sites according to standard protocol [17]. Participants also self-reported weight using the study-provided digital scales at 6, 12, and 18 months (if no study-measured weight). According to the trial protocol, in the case of missing study-measured weight, the closest weight measurement from the electronic health record within 3 months of the due date of a missed 18-month visit or self-reported weight (if no electronic health record weight) was used.

Session attendance was calculated for weekly sessions and monthly phone calls combined and separately in the videoconference and in-person groups, excluding the 15 participants who were transferred to the web-based video option. Session attendance was then compared between the videoconference and in-person groups using Student *t* tests.

All analyses were conducted using SAS version 9.4 (SAS Institute Inc). Statistical significance was defined as *P*<.05 (2-sided).

## Results

### Baseline Characteristics

As shown in Tables 2 and 3, the participants who attended were middle-aged (mean age 47.3, SD 11.8 years), educated (150/195, 76.9% attended at least some college), employed full- or part-time (167/194, 86.1%), and had access to a computer (165/184, 89.7%) and smartphone (179/184, 97.3%). Among these men, 28% (56/200) chose web-based videoconference groups at the orientation, 31% (62/200) chose web-based videos, and 41% (82/200) chose in-person groups. In addition, most preferred to engage in the intervention in English (142/200, 71%) versus Spanish (58/200, 29%).

**Table 2:** Baseline characteristics overall and by initial choice of intervention delivery (N=200).

Characteristic				All		Videoconference (n=56)		Web-based videos (n=62)		In-person group (n=82)		*P* value	
**Demographic**													
	Age (years), mean (SD)			47.3 (11.8)		45.6 (10.9)^a^		45.4 (11.4)^a^		50.0 (12.3)^b^		.03	
	**Income (US $; n=167), n (%)**												.02
		<75,000	49 (29.3)		8 (16)^a^		14 (25.9)^a,b^		27 (42.9)^b^				
		75,000-<150,000	53 (31.7)		17 (34)^a^		17 (31.5)^a,b^		19 (30.2)^b^				
		≥150,000	65 (38.9)		25 (50)^a^		23 (42.6)^a,b^		17 (27)^b^				
	**Education (n=195), n (%)**												.03
		High school, GED^c^, or less	45 (23.1)		7 (12.7)^a^		12 (19.7)^a,b^		26 (32.9)^b^				
		Some college	49 (25.1)		12 (21.8)^a^		19 (31.1)^a,b^		18 (22.8)^b^				
		College graduate	58 (29.7)		17 (30.9)^a^		21 (34.4)^a,b^		20 (25.3)^b^				
		More than college	43 (22.1)		19 (34.5)^a^		9 (14.8)^a,b^		15 (19)^b^				
	**Marital status (n=196), n (%)**												.07
		Married or living with a partner	156 (79.6)		38 (69.1)		52 (83.9)		66 (83.5)				
		Single, separated, divorced, or widowed	40 (20.4)		17 (30.9)		10 (16.1)		13 (16.5)				
	**Household size (n=191), n (%)**												.51
		1-2	13 (6.8)		4 (7.3)		4 (6.7)		5 (6.6)				
		3	35 (18.3)		10 (18.2)		10 (16.7)		15 (19.7)				
		4	44 (23)		19 (34.6)		10 (16.7)		15 (19.7)				
		5	49 (25.7)		10 (18.2)		18 (30)		21 (27.6)				
		≥6	50 (26.2)		12 (21.8)		18 (30)		20 (26.3)				
**Clinical**													
	BMI (kg/m^2^), mean (SD)			33.1 (5.2)		33.7 (5.7)		32.6 (5.4)		33.0 (4.6)		.48	
	Weight (kg), mean (SD)			101.5 (33.7)		102.7 (22.3)		96.7 (17.3)		104.3 (46.8)		.39	
	Waist circumference (cm), mean (SD)			109.5 (12.3)		111.0 (14.0)		106.8 (11.8)		110.6 (11.2)		.11	
	Number of metabolic risks, mean (SD)			2.0 (0.9)		1.9 (0.9)		2.0 (0.8)		2.0 (0.9)		.62	
	SBP^d^ (mm Hg), mean (SD)			122.4 (12.2)		121.2 (13.9)		121.5 (11.2)		123.9 (11.6)		.36	
	DBP^e^ (mm Hg), mean (SD)			78.6 (9.4)		78.1 (10.0)		79.2 (9.0)		78.5 (9.4)		.81	
	PHQ-9^f^ score (n=196), mean (SD)			3.5 (3.7)		3.6 (4.2)		3.6 (3.3)		3.2 (3.7)		.72	
	**EQ-5D-5L^g^** **: mobility (n=196), n (%)**												.63
		No problems	170 (86.7)		50 (90.9)		52 (83.9)		68 (86.1)				
		Slight problems	16 (8.2)		5 (9.1)		5 (8.1)		6 (7.6)				
		Moderate problems	8 (4.1)		0 (0)		4 (6.5)		4 (5.1)				
		Severe problems	2 (1)		0 (0)		1 (1.6)		1 (1.3)				
		Extreme problems	0 (0)		0 (0)		0 (0)		0 (0)				
	**EQ-5D-5L: self-care (n=196), n (%)**												.64
		No problems	193 (98.5)		54 (98.2)		62 (100)		77 (97.5)				
		Slight problems	2 (1)		1 (1.8)		0 (0)		1 (1.3)				
		Moderate problems	1 (0.5)		0 (0)		0 (0)		1 (1.3)				
		Severe problems	0 (0)		0 (0)		0 (0)		0 (0)				
		Extreme problems	0 (0)		0 (0)		0 (0)		0 (0)				
	**EQ-5D-5L: usual activities (n=196), n (%)**												.68
		No problems	171 (87.2)		50 (90.9)		51 (82.3)		70 (88.6)				
		Slight problems	19 (9.7)		4 (7.3)		8 (12.9)		7 (8.9)				
		Moderate problems	6 (3.1)		1 (1.8)		3 (4.8)		2 (2.5)				
		Severe problems	0 (0)		0 (0)		0 (0)		0 (0)				
		Extreme problems	0 (0)		0 (0)		0 (0)		0 (0)				
	**EQ-5D-5L: pain and discomfort (n=196), n (%)**												.15
		No problems	103 (52.6)		25 (45.5)		29 (46.8)		49 (62)				
		Slight problems	69 (35.2)		26 (47.3)		23 (37.1)		20 (25.3)				
		Moderate problems	20 (10.2)		4 (7.3)		8 (12.9)		8 (10.1)				
		Severe problems	4 (2)		0 (0)		2 (3.2)		2 (2.5)				
		Extreme problems	0 (0)		0 (0)		0 (0)		0 (0)				
	**EQ-5D-5L: anxiety and depression (n=196), n (%)**												.73
		No problems	147 (75)		40 (72.7)		46 (74.2)		61 (77.2)				
		Slight problems	35 (17.9)		9 (16.4)		12 (19.4)		14 (17.7)				
		Moderate problems	13 (6.6)		5 (9.1)		4 (6.5)		4 (5.1)				
		Severe problems	1 (0.5)		1 (1.8)		0 (0)		0 (0)				
		Extreme problems	0 (0)		0 (0)		0 (0)		0 (0)				
	Obesity-related problem raw score (n=196), mean (SD)			0.8 (0.7)		0.9 (0.8)		0.8 (0.7)		0.7 (0.7)		.29	
	PROMIS^h^ sleep disturbance T score (n=196), mean (SD)			47.3 (8.9)		47.7 (9.1)		48.8 (7.5)		45.8 (9.7)		.13	
	PROMIS sleep impairment T score (n=195), mean (SD)			47.5 (9.2)		48.4 (9.4)		48.2 (8.8)		46.3 (9.3)		.34	
**Employment**													
	**Employment status (n=194), n (%)**												.24
		Employed	167 (86.1)		51 (92.7)		55 (88.7)		61 (79.2)				
		Unemployed	5 (2.6)		1 (1.8)		1 (1.6)		3 (3.9)				
		Other (homemaker, student, retired, and disabled or not able to work)	22 (11.3)		3 (5.5)		6 (9.7)		13 (16.9)				
	**Work (hours per week; n=188), n (%)**												.18
		0 to <5	15 (8)		2 (3.8)		3 (4.9)		10 (13.5)				
		5 to <30	9 (4.8)		2 (3.8)		2 (3.3)		5 (6.8)				
		30 to <50	95 (50.5)		26 (49.1)		30 (49.2)		39 (52.7)				
		≥50	69 (36.7)		23 (43.4)		26 (42.6)		20 (27)				
	**Occupation (n=186), n (%)**												.04
		Mostly sitting or standing	128 (68.8)		44 (81.5)^a^		39 (67.2)^a,b^		45 (60.8)^b^				
		Mostly walking or heavy work	58 (31.2)		10 (18.5)^a^		19 (32.8)^a,b^		29 (39.2)^b^				
	**White- or blue-collar (n=152), n (%)**												.02
		White collar	108 (71.1)		39 (86.7)^a^		33 (68.8)^b^		36 (61)^b^				
		Blue collar	44 (29)		6 (13.3)^a^		15 (31.3)^b^		23 (39)^b^				
**Culture**													
	**Language^i^, n (%)**												<.001
		English	142 (71)		48 (85.7)^a^		61 (98.4)^j^		33 (40.2)^b^				
		Spanish	58 (29)		8 (14.3)^a^		1 (1.6)^j^		49 (59.8)^b^				
	Acculturation score, mean (SD)			3.4 (0.9)		3.7 (0.6)^a^		3.6 (0.7)^a^		3.1 (1.0)^b^		<.001	
	Health literacy score, mean (SD)			4.4 (1.9)		5.0 (1.5)^a^		4.5 (1.9)^a,b^		4.0 (2.0)^b^		.004	
	**Health literacy category, n (%)**												.02
		Adequate literacy	147 (73.5)		49 (87.5)^a^		47 (75.8)^a,b^		51 (62.2)^b^				
		Possibility of limited literacy	31 (15.5)		4 (7.1)^a^		8 (12.9)^a,b^		19 (23.2)^b^				
		High likelihood (≥50%) of limited literacy	22 (11)		3 (5.4)^a^		7 (11.3)^a,b^		12 (14.6)^b^				

^a^Different superscripts denote statistically significant differences.

^b^Different superscripts denote statistically significant differences.

^c^GED: General Educational Development.

^d^SBP: systolic blood pressure.

^e^DBP: diastolic blood pressure.

^f^PHQ-9: Patient Health Questionnaire-9.

^g^EQ-5D-5L: EuroQol 5-dimension 5-level.

^h^PROMIS: Patient-Reported Outcome Measurement System.

^i^Language that the patient preferred for the intervention.

^j^Different superscripts denote statistically significant differences.

**Table 3:** Baseline technology use and access overall and by initial choice of intervention delivery (N=184).

Characteristic			All, n (%)		Videoconference (n=54), n (%)		Web-based videos (n=55), n (%)		In-person group (n=75), n (%)		*P* value	
**Desktop or laptop computer**												.01
	Yes	165 (89.7)		54 (100)^a^		50 (90.9)^b^		61 (81.3)^b^				
	No	18 (9.8)		0 (0)^a^		5 (9.1)^b^		13 (17.3)^b^				
	Declined to state	1 (0.5)		0 (0)^a^		0 (0)^b^		1 (1.3)^b^				
**Smartphone (eg, iPhone or Android)**												.51
	Yes	179 (97.3)		54 (100)		53 (96.4)		72 (96)				
	No	4 (2.2)		0 (0)		2 (3.6)		2 (2.7)				
	Declined to state	1 (0.5)		0 (0)		0 (0)		1 (1.3)				
**Cell phone but not smartphone**												.43
	Yes	18 (9.8)		3 (5.6)		9 (16.4)		6 (8)				
	No	160 (87)		50 (92.6)		44 (80)		66 (88)				
	I’m not sure	1 (0.5)		0 (0)		0 (0)		1 (1.3)				
	Declined to state	5 (2.7)		1 (1.9)		2 (3.6)		2 (2.7)				
**Tablet (eg, iPad or Kindle)**												.07
	Yes	139 (75.5)		48 (88.9)		39 (70.9)		52 (69.3)				
	No	44 (23.9)		6 (11.1)		16 (29.1)		22 (29.3)				
	Declined to state	1 (0.5)		0 (0)		0 (0)		1 (1.3)				
**Dial-up internet service at home**												.21
	Yes	30 (16.3)		4 (7.4)		8 (14.6)		18 (24)				
	No	149 (81)		49 (90.7)		46 (83.6)		54 (72)				
	I’m not sure	1 (0.5)		0 (0)		0 (0)		1 (1.3)				
	Declined to state	4 (2.2)		1 (1.9)		1 (1.8)		2 (2.7)				
**Higher-speed broadband internet service such as DSL^c^, cable, or fiber optic service at home**												.09
	Yes	173 (94)		53 (98.2)		52 (94.6)		68 (90.7)				
	No	5 (2.7)		1 (1.9)		3 (5.5)		1 (1.3)				
	I’m not sure	5 (2.7)		0 (0)		0 (0)		5 (6.7)				
	Declined to state	1 (0.5)		0 (0)		0 (0)		1 (1.3)				
**How often do you use a computer at work, school, home, or anywhere else?**												.002
	Never	12 (6.5)		0 (0)^a^		2 (3.6)^b^		10 (13.3)^b^				
	Rarely	15 (8.2)		0 (0)^a^		6 (10.9)^b^		9 (12)^b^				
	Sometimes	7 (3.8)		0 (0)^a^		2 (3.6)^b^		5 (6.7)^b^				
	Often	26 (14.1)		7 (13)^a^		9 (16.4)^b^		10 (13.3)^b^				
	Very often	124 (67.4)		47 (87)^a^		36 (65.5)^b^		41 (54.7)^b^				
**How often do you communicate with others by email?**												.003
	Never	13 (7.1)		0 (0)^a^		4 (7.3)^a,b^		9 (12)^b^				
	Rarely	15 (8.2)		1 (1.9)^a^		2 (3.6)^a,b^		12 (16)^b^				
	Sometimes	31 (16.9)		7 (13)^a^		14 (25.5)^a,b^		10 (13.3)^b^				
	Often	29 (15.8)		11 (20.4)^a^		7 (12.7)^a,b^		11 (14.7)^b^				
	Very often	96 (52.2)		35 (64.8)^a^		28 (50.9)^a,b^		33 (44)^b^				
**How often do you talk to others using software or an app with video chat and voice call services?**												.01
	Never	38 (20.7)		7 (13)^a^		8 (14.6)^a,b^		23 (30.7)^b^				
	Rarely	44 (23.9)		7 (13)^a^		15 (27.3)^a,b^		22 (29.3)^b^				
	Sometimes	34 (18.5)		15 (27.8)^a^		9 (16.4)^a,b^		10 (13.3)^b^				
	Often	24 (13)		7 (13)^a^		7 (12.7)^a,b^		10 (13.3)^b^				
	Very often	43 (23.4)		18 (33.3)^a^		16 (29.1)^a,b^		9 (12)^b^				
	Declined to state	1 (0.5)		0 (0)^a^		0 (0)^a,b^		1 (1.3)^b^				
**How often do you access the internet on a cell phone, tablet, or other mobile handheld device?**												.22
	Never	6 (3.3)		0 (0)		2 (3.6)		4 (5.3)				
	Rarely	4 (2.2)		0 (0)		1 (1.8)		3 (4)				
	Sometimes	19 (10.3)		2 (3.7)		8 (14.6)		9 (12)				
	Often	22 (12)		6 (11.1)		5 (9.1)		11 (14.7)				
	Very often	132 (71.7)		46 (85.2)		39 (70.9)		47 (62.7)				
	Declined to state	1 (0.5)		0 (0)		0 (0)		1 (1.3)				
**How often do you use your cell phone to send or receive emails?**												.10
	Never	11 (6)		0 (0)		2 (3.6)		9 (12)				
	Rarely	20 (10.9)		8 (14.8)		2 (3.6)		10 (13.3)				
	Sometimes	30 (16.3)		8 (14.8)		11 (20)		11 (14.7)				
	Often	41 (22.3)		11 (20.4)		16 (29.1)		14 (18.7)				
	Very often	80 (43.5)		27 (50)		23 (41.8)		30 (40)				
	Declined to state	2 (1.1)		0 (0)		1 (1.8)		1 (1.3)				
**How often do you use your cell phone to send or receive SMS text messages?**												.31
	Never	4 (2.2)		0 (0)		2 (3.6)		2 (2.7)				
	Rarely	5 (2.7)		0 (0)		1 (1.8)		4 (5.3)				
	Sometimes	14 (7.6)		1 (1.9)		5 (9.1)		8 (10.7)				
	Often	36 (19.6)		12 (22.2)		10 (18.2)		14 (18.7)				
	Very often	122 (66.3)		41 (75.9)		36 (65.5)		45 (60)				
	Declined to state	3 (1.6)		0 (0)		1 (1.8)		2 (2.7)				
**How often do you use apps you downloaded to your mobile device (eg, smartphone or tablet)?**												.10
	Never	9 (4.9)		0 (0)		3 (5.5)		6 (8)				
	Rarely	16 (8.7)		5 (9.3)		2 (3.6)		9 (12)				
	Sometimes	32 (17.4)		6 (11.1)		14 (25.5)		12 (16)				
	Often	43 (23.4)		13 (24.1)		11 (20)		19 (25.3)				
	Very often	82 (44.6)		30 (55.6)		25 (45.5)		27 (36)				
	Declined to state	2 (1.1)		0 (0)		0 (0)		2 (2.7)				
**I feel completely comfortable using a web-based videoconferencing tool if someone shows me how to use it**												.13
	Strongly disagree	2 (1.1)		1 (1.9)		0 (0)		1 (1.3)				
	Disagree	8 (4.4)		1 (1.9)		3 (5.5)		4 (5.3)				
	Neither agree nor disagree	12 (6.5)		0 (0)		6 (10.9)		6 (8)				
	Agree	62 (33.7)		15 (27.8)		16 (29.1)		31 (41.3)				
	Strongly agree	99 (53.8)		37 (68.5)		30 (54.6)		32 (42.7)				
	Declined to state	1 (0.5)		0 (0)		0 (0)		1 (1.3)				
**I feel completely comfortable using smartphone apps to track my diet or physical activity if someone shows me how to use them**												.49
	Strongly disagree	5 (2.7)		1 (1.9)		2 (3.6)		2 (2.7)				
	Disagree	7 (3.8)		0 (0)		2 (3.6)		5 (6.7)				
	Neither agree nor disagree	14 (7.6)		2 (3.7)		4 (7.3)		8 (10.7)				
	Agree	58 (31.5)		16 (29.6)		18 (32.7)		24 (32)				
	Strongly agree	99 (53.8)		35 (64.8)		29 (52.7)		35 (46.7)				
	Declined to state	1 (0.5)		0 (0)		0 (0)		1 (1.3)				
**I feel completely comfortable watching web-based videos using my electronic device, such as laptop or tablet**												.12
	Strongly disagree	5 (2.7)		1 (1.9)		1 (1.8)		3 (4)				
	Disagree	5 (2.7)		0 (0)		1 (1.8)		4 (5.3)				
	Neither agree nor disagree	10 (5.4)		0 (0)		4 (7.3)		6 (8)				
	Agree	57 (31)		13 (24.1)		18 (32.7)		26 (34.7)				
	Strongly agree	106 (57.6)		40 (74.1)		31 (56.4)		35 (46.7)				
	Declined to state	1 (0.5)		0 (0)		0 (0)		1 (1.3)				

^a^Different superscripts denote statistically significant differences.

^b^Different superscripts denote statistically significant differences.

^c^DSL: digital subscriber line.

### Bivariate Associations

Participants in the 3 intervention delivery options had similar clinical characteristics (eg, BMI, blood pressure, depression symptoms, and overall and obesity-specific quality of life) but differed significantly according to demographic, employment, cultural, and technology use factors (Tables 2 and 3). Men who chose videoconference were more likely to be younger (*P*=.04), have higher income (*P*=.005) and education (*P*=.03), have a white-collar job (*P*=.004), prefer English (*P*<.001), be more acculturated (*P*<.001), have higher health literacy (*P*<.001), have access to a computer (*P*=.004), and have higher technology skills (eg, computer, *P*<.001; email, *P*=.003; and video chat app use, *P*=.002) compared with men who chose the in-person group. Similarly, men who chose web-based videos were more likely to be younger (*P*=.02) and more acculturated (*P*=.001) than those who chose the in-person group. They were also more likely to have a blue-collar job compared with those who chose videoconference (*P*=.04) and more likely to speak English than men who chose the videoconference (*P*=.01) or in-person (*P*<.001) groups. Men who chose web-based videos had intermediate technology skills compared with those who chose either the videoconference or in-person group and were less likely to have (*P*=.02) and use a computer (*P*=.02) than those who chose videoconference but as likely to use emails (*P*=.08) and video chat apps (*P*=.33) as men who chose videoconference.

### Multivariate Associations

Canonical discriminant analysis identified 1 orthogonal dimension representing statistically significant combinations of the baseline characteristics. The canonical variates of this single dimension explained 41% of the total variance of the choice of 3 intervention delivery options. Participants electing the in-person and web-based video options had the most extreme mean scores (0.98 vs −0.89; *P*<.001) on the canonical dimension 1. This signified that this dimension distinguished most significantly between these 2 intervention choices. According to characteristics with the highest positive or negative correlation coefficients and using 0.25 as a cutoff (Table 4), participants who were older, spoke Spanish, and did not use a computer frequently had a higher probability of choosing the in-person option versus the web-based videos option.

**Table 4:** Standardized coefficients from canonical discriminant analysis for individual baseline characteristics of participants in the HOMBRE (Hombres con Opciones para Mejorar su Bienestar para Reducir Enfermedades Crónicas) intervention (N=175)^a^.

Characteristic				Dimension 1^b^ coefficients
**Demographic**				
	Age		0.33	
	**Education (reference: high school, GED^c^, or less)**			
		Some college	0.03	
		College graduate	0.12	
		More than college	0.10	
	**Marital status (reference: single, separated, divorced, or widowed)**			
		Married or living with another person	−0.22	
**Clinical**				
	Waist circumference		0.11	
	Sleep disturbance T score		−0.20	
**Employment**				
	**Occupation (reference: no job)**			
		Mostly sitting or standing	0.23	
		Mostly walking or heavy work	0.15	
**Culture**				
	**Language (reference: English)**			
		Spanish	1.03	
	Short Acculturation Scale for Hispanics		0.09	
	Health literacy score		0.00	
**Technology use and access**				
	**If they had a desktop or laptop computer (reference: no)**			
		Yes	0.15	
	**If they had a tablet (eg, iPad or Kindle; reference: no)**			
		Yes	0.13	
	**If they had higher-speed broadband internet service such as DSL^d^, cable, or** **fiber optic service at home (reference: no)**			
		Yes	0.09	
	**How often do you use a computer at work, school, home, or anywhere else?** **(reference: never, rarely, or sometimes)**			
		Often or very often	−0.27	
	**How often do you communicate with others by email? (reference: never, rarely,** **or sometimes)**			
		Often or very often	−0.08	
	**How often do you talk to others using software or an app with video chat and** **voice call services? (reference: never, rarely, or sometimes)**			
		Often or very often	−0.06	
	**How often do you use your cell phone to send or receive emails?** **(reference: never, rarely, or sometimes)**			
		Often or very often	0.03	
	**How often do you use apps you downloaded on your mobile device** **(eg, smartphone or tablet; reference: never, rarely, or sometimes)?**			
		Often or very often	−0.01	
	**I feel completely comfortable using a web-based videoconferencing tool if** **someone shows me how to use it (reference: neither agree nor disagree, disagree,** **or strongly disagree).**			
		Agree or strongly agree	−0.08	
	**I feel completely comfortable watching web-based videos using my electronic** **device, such as laptop or tablet (reference: neither agree nor disagree, disagree,** **or strongly disagree).**			
		Agree or strongly agree	0.13	

^a^Results based on 175 HOMBRE intervention participants who had complete data for all baseline characteristics used in the canonical discriminant analysis.

^b^Dimension 1: canonical function *F*_44,302_=2.82 (*P*<.001); *R*^2^ of the canonical correlation=0.41.

^c^GED: General Educational Development.

^d^DSL: digital subscriber line.

### Weight Loss

Compared with men who initially chose web-based videos, those who initially chose videoconference and in-person group sessions lost significantly more weight at 6 months (mean −3.9, SD 6.1 kg for videoconference and mean −4.3, SD 5.3 kg for in-person vs mean −0.3, SD 3.7 kg for web-based videos; *P*<.001 for both comparisons) and 18 months (mean −3.8, SD 8.4 kg for videoconference and mean −3.3, SD 6.0 kg for in-person vs mean −0.9, SD 4.6 kg for web-based videos; *P*=.02 and *P*=.04, respectively, for the comparisons), and those who chose in-person group sessions also lost significantly more weight at 12 months (mean −4.1, SD 6.0 kg vs mean −1.0, SD 4.8 kg; *P*=.008; Table 5). After accounting for men who were transferred to the web-based videos group at 4 weeks into the intervention after not attending group sessions in the videoconference (11/15, 73%) and in-person (4/15, 27%) group sessions, men in both of the group session formats (videoconference and in-person) lost significantly more weight than men in the web-based videos group at 6, 12, and 18 months.

**Table 5:** Weight change by initial and final choice of intervention delivery (N=200).

Weight change^a^	Initial intervention choice, mean (SD)				Final intervention choice, mean (SD)		
	Videoconference (n=56)	Web-based videos (n=62)	In-person group (n=82)	Videoconference (n=45)		Web-based videos (n=77)	In-person group (n=78)
Weight change at 6 months from baseline (kg)	−3.9 (6.1)^b^	−0.3 (3.7)	−4.3 (5.3)^b^	−4.8 (5.7)^b^		−0.4 (4.3)	−4.4 (5.3)^b^
Weight change at 12 months from baseline (kg)	−3.4 (7.9)	−1.0 (4.8)	−4.1 (6.0)^c^	−4.5 (8.0)^c^		−0.6 (4.8)	−4.2 (6.1)^c^
Weight change at 18 months from baseline (kg)	−3.8 (8.4)^d^	−0.9 (4.6)	−3.3 (6.0)^d^	−4.8 (8.6)^c^		−0.8 (4.6)	−3.2 (6.2)^d^

^a^Electronic health record–abstracted and self-reported weights were used for 6 and 12 months, and study-measured, electronic health record–abstracted, and self-reported weights were used for 18 months.

^b^Significant difference at *P*<.001 for comparing with the web-based video group.

^c^Significant difference at *P*<.01 for comparing with the web-based video group.

^d^Significant difference at *P*<.05 for comparing with the web-based video group. There was no difference between the in-person and videoconference groups at all time points.

### Session Attendance

There was no significant difference (*P*>.05) in session attendance between the videoconference and in-person groups (Table 6). Overall, in both groups (excluding those who transferred to the web-based video option), the mean number of sessions attended was 16.5 (SD 6.5), with 86.9% (107/123) attending >25% sessions, 83.7% (103/123) attending >50% sessions, and 73.2% (90/123) attending >75% of the 21 sessions (1 orientation, 12 weekly sessions, and 8 monthly phone calls). Of the 12 weekly sessions, the participants attended an average of 10.1 (SD 3.8) sessions. Of the 8 monthly phone calls, they completed an average of 5.4 (SD 3.1) calls.

**Table 6:** Session attendance among participants in the web-based and in-person groups^a^ (N=123).

Group	Number of total sessions attended (including makeup sessions, out of 21 sessions), mean (SD)	*P* value	Number of weekly core sessions attended (including makeup sessions, out of 12 sessions), mean (SD)	*P* value	Number of monthly contacts received (including makeup sessions, out of 8 calls), mean (SD)	*P* value
Overall (in person and videoconference)	16.5 (6.5)	N/A^b^	10.1 (3.8)	N/A	5.4 (3.1)	N/A
Videoconference	17.9 (4.7)	.07	10.9 (2.6)	.07	5.9 (2.6)	.13
In person	15.7 (7.3)	.07	9.7 (4.3)	.07	5.1 (3.3)	.13

^a^A total of 15 participants who were transferred to the web-based video option after not attending in-person or videoconference sessions were excluded.

^b^N/A: not applicable.

## Discussion

### Principal Findings

This study found that, when provided with a choice on how to engage in a weight loss intervention, 28% (56/200) of men chose videoconference groups, 31% (62/200) chose web-based videos, and 41% (82/200) chose in-person groups. There were significant differences in demographic, employment, cultural, and technology use and access factors among men who chose 1 of the 3 different options. For example, men who chose web-based videoconference groups were more likely to be younger, have higher income and education, have a white-collar type of job, prefer English, be more acculturated, have higher health literacy, have access to a computer, and have higher technology skills (eg, computer, email, and video chat app use) compared with men who chose in-person groups. Our multivariate analysis distinguished most significantly between those who chose in-person groups versus those who chose web-based videos. Men who were older, spoke Spanish, and did not use a computer frequently had a higher probability of choosing in-person groups versus web-based videos. In terms of weight loss, men who chose the videoconference and in-person group sessions lost more weight than those who chose web-based videos.

### Comparison With Prior Work

Similar to this study, Piatt et al [33] tested 3 GLB-based lifestyle intervention delivery options (in-person groups, DVD, and internet education) among 555 adults (95% were White) and compared them with 1 arm where participants could choose the option (the preference arm). Their in-person groups and DVD options were similar to those offered in this study. In the preference arm, 60% chose the in-person option, none chose the DVD option, and 40% chose the internet education option. In our study, 41% (82/200) chose the in-person group, 31% (62/200) chose the web-based videos group, and 28% (56/200) chose the videoconference group. This difference may be attributed to the differences in sample demographics between the 2 studies—mean age of 52.3 (SD 12.7) years in the study by Piatt et al [33] versus 47.3 (SD 11.8) years in our study, 97% non-Latino White versus 100% (200/200) Latino sample, and 97% women versus 100% (200/200) men. Piatt et al [33] did not report on weight loss for each option but found that participants who were given a choice among the 3 options lost more weight at 6 months (−6.4 kg) than those who were randomly assigned to one of the options without a choice (−5.7 kg for in-person groups, −5.5 kg for DVD, and −6.2 kg for internet education; *P*<.001). In addition, Piatt et al [33] reported that the average session attendance was 8.4 for the preference arm participants who chose in-person groups and 9.2 for the preference arm participants who chose internet education. Similar to the study by Piatt et al [33], group session attendance in our study did not differ according to whether men initially chose the videoconference (mean 14.9, SD 7.4 sessions) or in-person (mean 15.0, SD 7.7 sessions) group. This could be because of the effectiveness of the protocol for supporting men in making a choice. The protocol included an explanation of the options from a health coach, included family members, and included peer discussion of the options. It is also possible that men could make an informed choice with less support, such as from a provider or staff member in the primary care setting. Future research could examine the most efficient and effective strategy for supporting men in making an informed choice about technology-mediated interventions.

### Implications

These findings have important implications for translation to practice, especially given the COVID-19 pandemic, which has made technology-mediated options necessary in the short term and possibly more common in the long term. The fact that 59% (118/200) of Latino men in this study preferred a technology-mediated option (ie, videoconference or web-based videos) suggests that these can be acceptable delivery formats for chronic disease prevention programs for this important high-risk population. This is consistent with previous literature indicating that Latinos are comfortable with technology and open to technology-mediated interventions [34-36]. However, given that the web-based videos were less effective for weight loss than either of the group-based options, future research should focus on increasing the effectiveness of web-based video options. In addition, including the option of in-person interventions appears to be important for reaching other subgroups of Latino men, such as those with lower health literacy and limited access to technology. Recognizing the diversity of Latino men and understanding the specific sociodemographics is critical to designing effective and engaging interventions to reach this population. As in the HOMBRE intervention, offering a suite of choices may be important for reaching the diversity of Latino men.

These findings demonstrate that Latino men from lower socioeconomic backgrounds may need additional support for accessing and using technology-mediated interventions. Technology use and access differ along socioeconomic lines in the United States, including among Latinos, with lower socioeconomic-level subgroups having less reliable access to technology than their higher socioeconomic-level counterparts [37,38]. Additional support could include resources such as the provision of computers, tablets, or smartphones and reliable internet access for delivering technology-mediated interventions. Resources could also include capacity building in the specific technologies used in the intervention. Future research can test strategies to increase access to and familiarity with technology-mediated interventions for Latino men from lower socioeconomic backgrounds. Furthermore, the clinics that serve Latino men who may need additional support to access technology-mediated interventions tend to also be underresourced. Thus, policies that provide additional funding and support to enable these clinics to implement technology-mediated interventions will be needed. This is especially important during the COVID-19 pandemic or other circumstances when technology-mediated interventions are needed.

The findings from the HOMBRE trial may be specific to the type of technology-mediated interventions offered. The 2 technology-mediated options included smartphone apps for tracking diet and physical activity and either videoconferencing or web-based videos for the intervention sessions. Preferences may vary according to the type of technology used in the intervention. Other technologies such as SMS text messaging, social media, and digital voice assistants may be appealing to other groups of Latino men. For example, Latino migrant farm workers have high (81%-97%) access to mobile phones and prefer talking and SMS text messaging using their phones [36,39]. Therefore, behavioral interventions using mobile phones and SMS text messages may be more acceptable to this population than those using computers or laptops. Latinos are also highly engaged in social media (73% of adult internet users use Facebook, 34% use Instagram, and 25% use Twitter) [40], which could be leveraged for behavioral lifestyle interventions.

### Limitations

This study has several limitations. First, all the technology use data were self-reported. Participants may have reported answers regarding their prior and current use of technology that they may have perceived as desirable [41]. Second, this study was conducted among a diverse sample of Latino men in the San Francisco Bay Area of California. The results of this investigation may not be generalizable to Latino men residing in other parts of the United States. Nevertheless, the findings appear to be consistent with previous findings on Latinos. Replication and assessment of generalizability in independent samples in other parts of the United States are needed to generalize the findings to a broader population. Third, we were not able to capture the frequency of videos viewed by the group that chose web-based videos. This would have enabled us to compare engagement in the intervention with the other choices. Fourth, we did not collect information on family member involvement, precluding an analysis of differences in family member involvement across delivery options. Fifth, caution should be used when interpreting the differences in weight loss among the intervention options, given that men were not randomized into these options. It is possible that the men who chose the videoconference and in-person groups were more motivated to lose weight than those who chose the web-based videos. Related, we were not able to determine the extent to which different levels of coach and peer support contributed to weight loss differences among the intervention options. The videoconference and in-person groups had the highest levels of coach and peer support. Future studies could examine the effectiveness of different delivery options and what aspects of these delivery options (eg, level of coach and peer support [42]) contribute to the varied effectiveness.

### Conclusions

In conclusion, this study revealed that among a diverse group of Latino men recruited from primary care, 28% (56/200) chose videoconference groups, 31% (62/200) chose web-based videos, and 41% (82/200) chose in-person groups. Differences in demographic, employment, cultural, and technology use factors distinguished between men who chose each of the options, suggesting that when offering interventions in diverse groups of Latino men, choice of delivery may be recommended. We also found that men attending either of the group-based options (videoconference and in-person) lost more weight than men who chose the web-based videos.

## Abbreviations

GLB: Group Lifestyle Balance
HOMBRE: Hombres con Opciones para Mejorar su Bienestar para Reducir Enfermedades Crónicas
